# Targeting of the HER2/HER3 signaling axis overcomes ligand‐mediated resistance to trastuzumab in HER2‐positive breast cancer

**DOI:** 10.1002/cam4.1995

**Published:** 2019-01-31

**Authors:** Satomi Watanabe, Kimio Yonesaka, Junko Tanizaki, Yoshikane Nonagase, Naoki Takegawa, Koji Haratani, Hisato Kawakami, Hidetoshi Hayashi, Masayuki Takeda, Junji Tsurutani, Kazuhiko Nakagawa

**Affiliations:** ^1^ Department of Medical Oncology Kindai University Faculty of Medicine Osaka‐Sayama, Osaka Japan; ^2^ Advanced Cancer Translational Research Institute, Showa University Tokyo Japan

**Keywords:** heregulin (HRG), human epidermal growth factor receptor 2 (HER2), human epidermal growth factor receptor 3 (HER3), neuregulin (NRG), patritumab (U3‐1287)

## Abstract

HER2‐targeted therapy, especially the anti‐HER2 antibody trastuzumab, is standard for HER2‐positive breast cancer; however, its efficacy is limited in a subpopulation of patients. HER3 ligand (heregulin)‐dependent HER2‐HER3 interactions play a critical role in the evasion of apoptosis and are therefore a target for oncotherapy to treat HER2‐positive breast cancer. The anti‐HER2 antibody pertuzumab and anti‐HER3 antibody patritumab both target this heregulin–HER3‐HER2 complex in different ways. This study examined the anticancer efficacy of dual HER2 and HER3 blockade in trastuzumab‐resistant HER2‐positive breast cancer. HER2‐positive SKBR3 or BT474 cells overexpressing heregulin (SKBR3‐HRG, BT474‐HRG) were used to evaluate the efficacy of trastuzumab, pertuzumab, and patritumab in vitro by performing cell viability, immunoblotting, and clonogenic assays. The effects of these agents were then evaluated in vivo using BT474‐HRG and an intrinsic heregulin‐expressing and HER2‐positive JIMT‐1 xenograft models. SKBR3‐HRG and BT474‐HRG cells lost sensitivity to trastuzumab, which was accompanied by Akt activation. Unexpectedly, trastuzumab in combination with pertuzumab or patritumab also showed limited efficacy toward these cells. In contrast, trastuzumab/pertuzumab/patritumab triple treatment demonstrated potent anticancer efficacy, concomitant with strong repression of Akt. Finally, in heregulin‐expressing BT474‐HRG and JIMT‐1 xenograft models, the addition of pertuzumab and patritumab to trastuzumab also enhanced antitumor efficacy leading to tumor regression. The current study found that triple blockade of HER2 and HER3 using trastuzumab, pertuzumab, and patritumab could overcome resistance to trastuzumab therapy in heregulin‐expressing and HER2‐positive breast cancer, which could be exploited clinically.

## 1 INTRODUCTION

Breast cancer is the leading cause of cancer‐related death among females worldwide and its incidence is increasing in Asian countries including Japan, Korea, and China.^1^ Treatment for advanced breast cancer depends on tumor characteristics. Approximately, 15%‐25% of breast cancers consist of human epidermal growth factor 2 (HER2)‐overexpressing or *HER2* amplified types.^2^ HER2‐targeted therapy, especially the anti‐HER2 antibody trastuzumab, has become the standard treatment for HER2‐positive breast cancer.^3^ Furthermore, another anti‐HER2 antibody pertuzumab was shown to significantly improve survival in patients with metastatic HER2‐positive breast cancer when combined with trastuzumab and docetaxel.^4^, ^5^ Pertuzumab can interrupt heterodimerization between HER2 and HER3, thereby preventing downstream signaling.

HER3, a HER family member, is aberrantly expressed in breast cancer.^6^ Due to structural features and its lack of intrinsic kinase activity, it cannot be autophosphorylated, but can be transphosphorylated through heterodimerization with other HER family members, especially HER2.^7^ The HER3 ligand heregulin activates HER3 and its downstream phosphoinositide 3‐kinase (PI3K)/AKT antiapoptotic signaling pathway through autocrine or paracrine mechanisms.^8^, ^9^, ^10^ Previously, we reported that heregulin mediates resistance to EGFR inhibitors in nonsmall cell lung and colorectal cancers.^11^, ^12^, ^13^ Furthermore, heregulin‐expressing HER2‐positive breast and gastric cancers exhibit heterogeneous susceptibility to anti‐HER2 agents like trastuzumab, lapatinib, and T‐DM1.^14^

Based on its critical role in cancer cell survival, HER3 is considered a promising onco‐therapeutic target.^7^, ^10^ Accordingly, multiple monoclonal antibodies targeting this receptor have been investigated preclinically and clinically.^12^, ^15^, ^16^, ^17^ One of these anti‐HER3 antibodies, patritumab, is a full human monoclonal antibody directed against the extracellular domain of HER3.^12^ We reported that its efficacy depends on heregulin level in cancer cells, as is generally observed for anti‐HER3 agents.^12^, ^15^, ^16^ Furthermore, some studies on anti‐HER3 antibodies such as seribantumab demonstrated their efficacy in patients with heregulin‐positive cancer; however, other clinical trials on anti‐HER3 antibodies did not report obvious relationships between efficacy and heregulin levels.^7^, ^18^, ^19^, ^20^ Therefore, the clinical relationship between anti‐HER3 efficacy and heregulin expression is still controversial.

Alternatively, to optimize anti‐HER3 therapeutics, agents for optimal combination therapy must be uncovered. The current investigation examined the combination of the anti‐HER3 antibody patritumab with the anti‐HER2 antibody pertuzumab for HER2‐positive breast cancer. Both drugs target ligand‐dependent HER3 activation in cancer in different manners. Specifically, pertuzumab inhibits ligand‐dependent HER2/HER3 dimerization, whereas patritumab binds the extracellular domain of HER3, presumably preventing heregulin binding. Considering these unique mechanisms, we hypothesized that heregulin could alter the efficacy of HER2‐ or HER3‐targeting drugs, and that the combined use of pertuzumab and patritumab could suppress cancer cell proliferation, better than each antibody alone, in heregulin‐expressing breast cancer.

## 2 MATERIALS AND METHODS

### 2.1 Cells and reagents

Human SKBR3, BT474, MDA‐MB‐453, and HCC1419 cell lines were obtained from the American Type Culture Collection (ATCC; Manassas, VA). The human JIMT‐1 cell line was obtained from Deutsche Sammlung von Mikroorganismen und Zellkulturen GmbH (DSMZ; Braunschweig, Germany). SKBR3 cells transfected with heregulin (SKBR3‐HRG) and SKBR3 cells transfected with the corresponding empty vector (SKBR3‐Mock) were previously established.^14^ We also established BT474‐HRG and BT474‐Mock cells transfected with heregulin or empty vector, respectively, as previously described.^14^ Cells were maintained in a humidified atmosphere of 5% CO_2_/95% air at 37°C in Roswell Park Memorial Institute 1640 medium (for JIMT‐1 cells) or Dulbecco's Modified Eagle Medium containing 10% fetal bovine serum (FBS). Cells were routinely tested for mycoplasma using MycoAlert (LT07; Lonza, Basel, Switzerland) and were negative. Trastuzumab and pertuzumab were purchased from Chugai Pharmaceuticals (Tokyo, Japan). Patritumab was provided by Daiichi‐Sankyo Co., Ltd. (Tokyo, Japan).

### 2.2 Cell viability assay

Cells were transferred to 96‐well flat‐bottomed plates and cultured overnight before exposure to various concentrations of trastuzumab, pertuzumab, and patritumab in medium containing 2% FBS for 120 hours. Cell Counting Kit‐8 solution (CK04; Dojindo, Kumamoto, Japan) was added to each well and cells were incubated for 3 hours at 37°C before measuring absorbance values using a Multiskan Spectrum instrument (Thermo Fisher Scientific, Waltham, MA). Values are expressed as the percentage of absorbance relative to that for untreated cells. The combination index (CI) was calculated using CalcuSyn v.2.1 software (Biosoft, Cambridge, UK). Values <1, = 1, and >1 indicated synergistic, additive, and antagonistic effects, respectively.

### 2.3 Immunoblotting

Cells were washed twice with ice‐cold phosphate‐buffered saline (PBS) and lysed with 1× Cell Lysis Buffer (Cell Signaling Technology, Danvers, MA) composed of 20 mmol/L tris‐HCl (pH 7.5), 150 mmol/L NaCl, 1 mmol/L EDTA (disodium salt), 1 mmol/L EGTA, 1% triton X‐100, 2.5 mmol/L sodium pyrophosphate, 1 mmol/L β‐glycerophosphate, 1 mmol/L Na_3_VO_4_, 1 μg/mL leupepsin, and 1 mmol/L phenylmethylsulfonyl fluoride. Lysate protein concentrations were determined using a bicinchoninic acid assay kit (Thermo Fisher Scientific) and equal amounts of protein were subjected to sodium dodecyl sulfate‐polyacrylamide gel electrophoresis on a 7.5% gel to analyze intracellular signaling or a 12% gel to analyze apoptosis (Bio‐Rad, Hercules, CA). Separated proteins were transferred to a nitrocellulose membrane, which was incubated with Blocking One or Blocking One‐P (for phosphorylated proteins) solution (both from Nacalai Tesque, Kyoto, Japan) for 20 minutes at room temperature before overnight incubation at 4°C with primary antibodies against phosphorylated HER2 (phospho [p]‐Tyr1248; #2247), p‐HER3 (Tyr1289; #4791), Akt (#9272), p‐Akt (Ser473; #9271), Erk (#9102), or heregulin (#2573), all from Cell Signaling Technology. Anti‐HER2 (06‐562) antibody was from Millipore (Bedford, MA). Antibodies against HER3 (sc‐285) and p‐Erk (Thr202/Tyr204; sc‐16982) were from Santa Cruz Biotechnology (Santa Cruz, CA) and the antibody against β‐actin (#10021) was from Sigma‐Aldrich (St. Louis, MO). Membranes were washed with PBS containing 0.05% tween 20 before incubating them for 2 hours at room temperature with horseradish peroxidase‐conjugated secondary antibodies (NA934; GE Healthcare, Indianapolis, IL). Immune complexes were detected with enhanced chemiluminescence reagent (RPN3244; GE Healthcare). Quantification was conducted using ImageQuant TL ver.8.1 (GE Healthcare).

### 2.4 Clonogenic assays

Cells were harvested, plated in 6‐well plates, and cultured in a medium containing 10% FBS. Cells were then incubated for 6 hours in an incubator and allowed to attach to the plates. Each drug was then added at a final concentration of 50 μg/mL. Cells were incubated until those in control plates become confluent. Media and drugs were changed every 5 days. After 26, 14, and 17 days of incubation for SKBR3‐Mock, SKBR3‐HRG, and BT474‐HRG cells, respectively, plates were gently washed with PBS and fixed with fixation solution (acetic acid/methanol 1:7) for 5 minutes. Plates were then rinsed again with PBS and colonies were stained with 0.5% crystal violet at room temperature for 2 hours. After staining, plates were immersed in tap water to rinse off excess stain. The percent colony area was automatically calculated using an ImageJ Plugin after image acquisition as described.^21^

### 2.5 Mouse xenograft studies

BT474‐HRG cells (1 × 10^7^ per mouse) and JIMT‐1 cells (5 × 10^6^ per mouse) suspended in 50% Matrigel in PBS were subcutaneously injected into the flank of 7‐week‐old female athymic nude mice (BALB/cAJcl‐*nu*/*nu*) obtained from CLEA Japan (Tokyo, Japan). Mice were divided into six treatment groups and treatments were initiated when the smallest tumors in each group reached 100 mm^3^. For 3 weeks, mice were intraperitoneally administered weekly doses of vehicle (PBS), trastuzumab (10 mg/kg), pertuzumab (25 mg/kg), patritumab (25 mg/kg), pertuzumab (25 mg/kg) + patritumab (25 mg/kg), or trastuzumab (10 mg/kg) + pertuzumab (25 mg/kg) + patritumab (25 mg/kg). The control group comprised six mice, whereas treatment groups consisted of seven mice each. Tumor volume was determined based on caliper measurements of tumor length (L) and width (W) according to the formula LW^2^/2. Tumor size and body weight were measured twice weekly. Mice were sacrificed at the end of the treatment period and tumor tissue was flash frozen at −80°C for immunoblotting. Animal experiments were performed in accordance with the Recommendations for Handling of Laboratory Animals for Biomedical Research compiled by the Committee on Safety and Ethical Handling Regulations or Laboratory Animal Experiments of Kindai University. The study was also reviewed and approved by the Animal Ethics Committee of Kindai University.

### 2.6 Statistical analysis

Quantitative data are presented as mean ± SE, unless otherwise indicated. Data were analyzed by performing two‐sided unpaired *t* tests for clonogenic assays and two‐way analysis of variance (ANOVA) for in vivo studies using GraphPad Prism v.7 software (GraphPad Inc, La Jolla, CA). A *P* value <0.05 was considered statistically significant.

## 3 RESULTS

### 3.1 Heregulin mediates trastuzumab resistance in HER2‐positive SKBR3 and BT474 breast cancer cell lines

**Figure 1:**
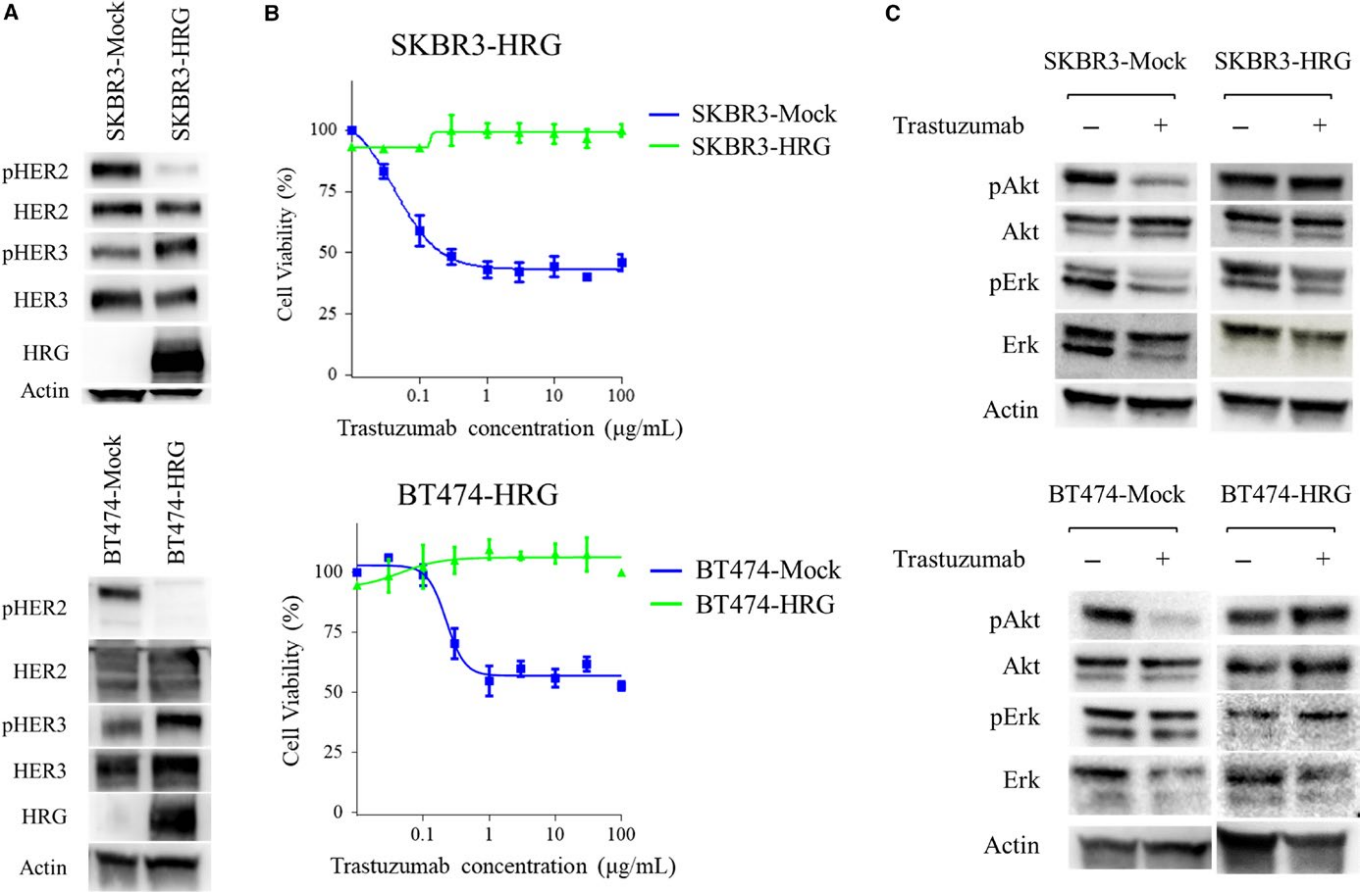
Heregulin‐expressing cells exhibit resistance to single‐agent trastuzumab therapy. A, SKBR3‐Mock, SKBR3‐HRG, BT474‐Mock, and BT474‐HRG cells were incubated for 24 h in RPMI containing 2% FBS, and then cell lysates were analyzed for the expression of pHER2, HER2, pHER3, HER3, and heregulin (HRG), with β‐actin serving as a loading control. B, SKBR3‐Mock, SKBR3‐HRG, BT474‐Mock, and BT474‐HRG cells were incubated for 120 h with increasing concentrations of trastuzumab; cell viability was then assessed with the Cell Counting Kit‐8. Each point represents the mean ± standard error of three independent experiments. C, SKBR3‐Mock, SKBR3‐HRG, BT474‐Mock, and BT474‐HRG cells were incubated for 24 h in RPMI containing 2% FBS, after which cells were treated with or without trastuzumab (20 μg/mL) for 1 h. Then, the cells were lysed and subjected to immunoblot analysis for pAkt, Akt, pErk, and Erk, with β‐actin serving as a loading control

The human breast cancer cell lines, SKBR3 and BT474, overexpress HER2 and are sensitive to trastuzumab. A heregulin‐overexpression plasmid was transfected into both lines to establish SKBR3‐HRG or BT474‐HRG cell lines, respectively.^14^ SKBR3‐Mock and BT474‐Mock cell lines were developed by transfecting SKBR3 or BT474 cells with the corresponding empty vector. Immunoblotting analysis demonstrated overexpression of heregulin, upregulation of pHER3, and downregulation of pHER2 in heregulin‐transfected cells compared to those in Mock‐transfected cells (Figure 1A). Consistent with our previous report, trastuzumab decreased control cell viability, which was accompanied by the degradation of downstream molecules, specifically pAkt in both SKBR3‐Mock and BT474‐Mock cells and pErk in SKBR3‐Mock cells (14). However, SKBR3‐HRG and BT474‐HRG cells were resistant to trastuzumab, and maintained Akt and Erk activation despite trastuzumab exposure (Figure 1B,C). These observations suggested that trastuzumab does not sufficiently inhibit Akt activation during heregulin‐dependent signaling. Therefore, heregulin was thought to mediate trastuzumab resistance in HER2‐positive breast cancer cells.

### 3.2 The addition of pertuzumab or patritumab to trastuzumab is not sufficient to overcome heregulin‐mediated resistance

**Figure 2:**
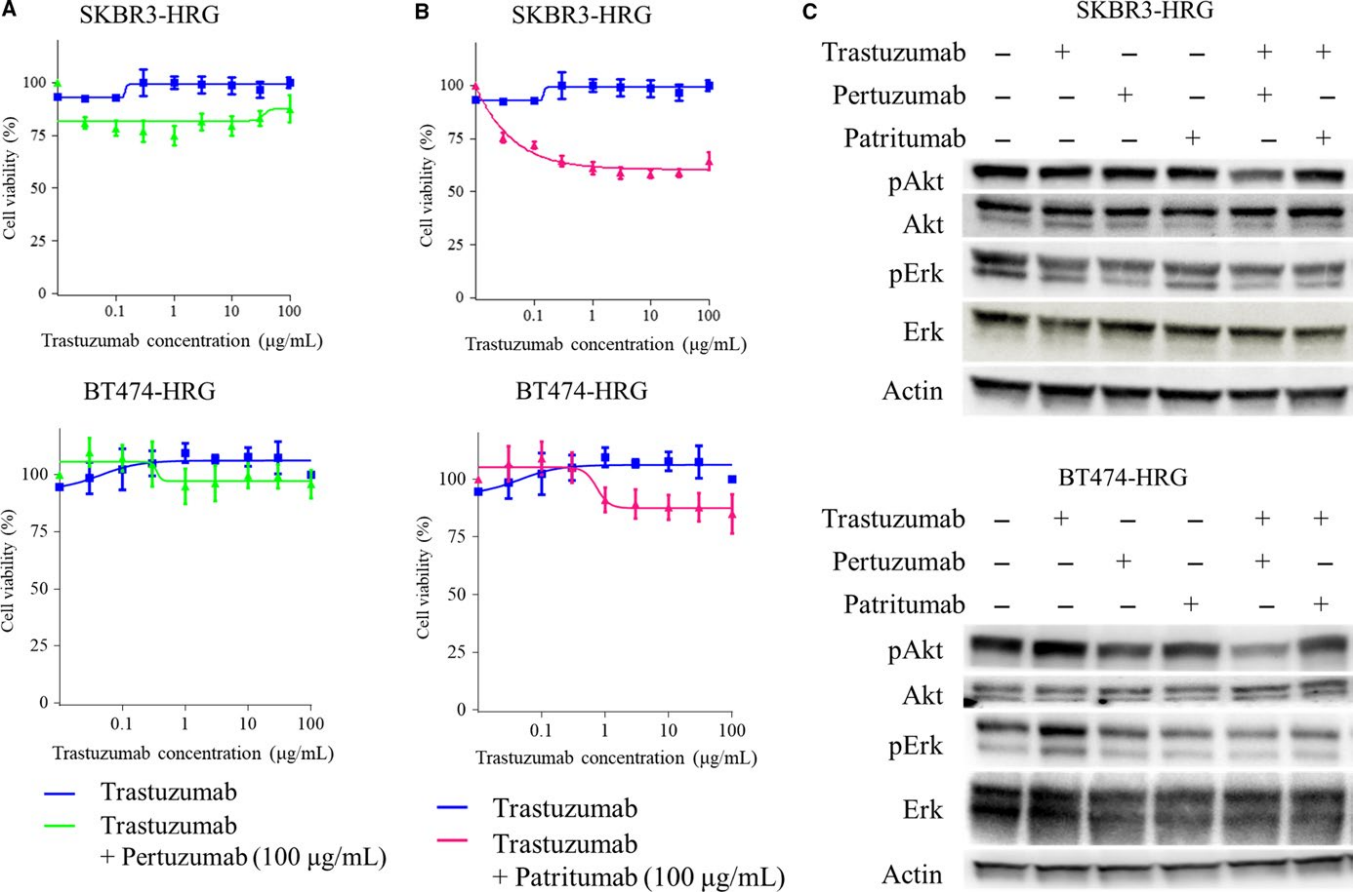
Addition of pertuzumab or patritumab to trastuzumab does not restore sensitivity in heregulin‐expressing cells. A and B, SKBR3‐HRG and BT474‐HRG cells were incubated for 120 h with increasing concentrations of trastuzumab and a fixed dose of pertuzumab (100 μg/mL) (A) or patritumab (100 μg/mL) (B); cell viability was then assessed with Cell Counting Kit‐8. Each point represents the mean ± standard error of 6‐wells from three independent experiments. C, SKBR3‐HRG and BT474‐HRG cells were incubated for 24 h in RPMI containing 2% FBS, after which cells were treated with or without trastuzumab (20 μg/mL), pertuzumab (20 μg/mL), and patritumab (20 μg/mL) for 1 h. Then, cells were lysed and subjected to immunoblot analysis for pAkt, Akt, pErk, and Erk, with β‐actin serving as a loading control

Next, we examined the anticancer efficacy of combined HER‐family‐targeting by performing cell viability assays. A previous clinical trial demonstrated that adding the anti‐HER2 antibody pertuzumab could improve overall survival compared to that with trastuzumab plus docetaxel in HER2‐positive metastatic breast cancer.^4^, ^5^ Unexpectedly, the addition of pertuzumab to trastuzumab did not restore or minimally restored sensitivity in heregulin‐expressing SKBR3‐HRG or BT474‐HRG cells (Figure 2A). Second, since heregulin‐expressing cancer cells were sensitive to several anti‐HER3 antibodies including patritumab, we examined whether this agent could overcome heregulin‐mediated resistance.^12^, ^15^, ^16^ The combination of patritumab and trastuzumab inhibited cell viability compared to that with trastuzumab alone; however, the viability curve did not reach the IC_50_, indicating that this combination was still not sufficient to inhibit heregulin‐expressing cell growth (Figure 2B). Consistent with this observation, pertuzumab monotherapy, patritumab monotherapy, or pertuzumab/trastuzumab and patritumab/trastuzumab combination therapy did not sufficiently decrease Akt or Erk phosphorylation in these cells (Figure 2C). This suggested that patritumab or pertuzumab alone cannot block Akt activation in heregulin‐expressing, HER2‐positive breast cancer. Therefore, pertuzumab/trastuzumab or patritumab/trastuzumab combination does not overcome heregulin‐mediated trastuzumab resistance.

### 3.3 Triple combination trastuzumab, pertuzumab, and patritumab therapy demonstrates potent anticancer effects by inhibiting Akt signaling in heregulin‐expressing cancer

**Figure 3:**
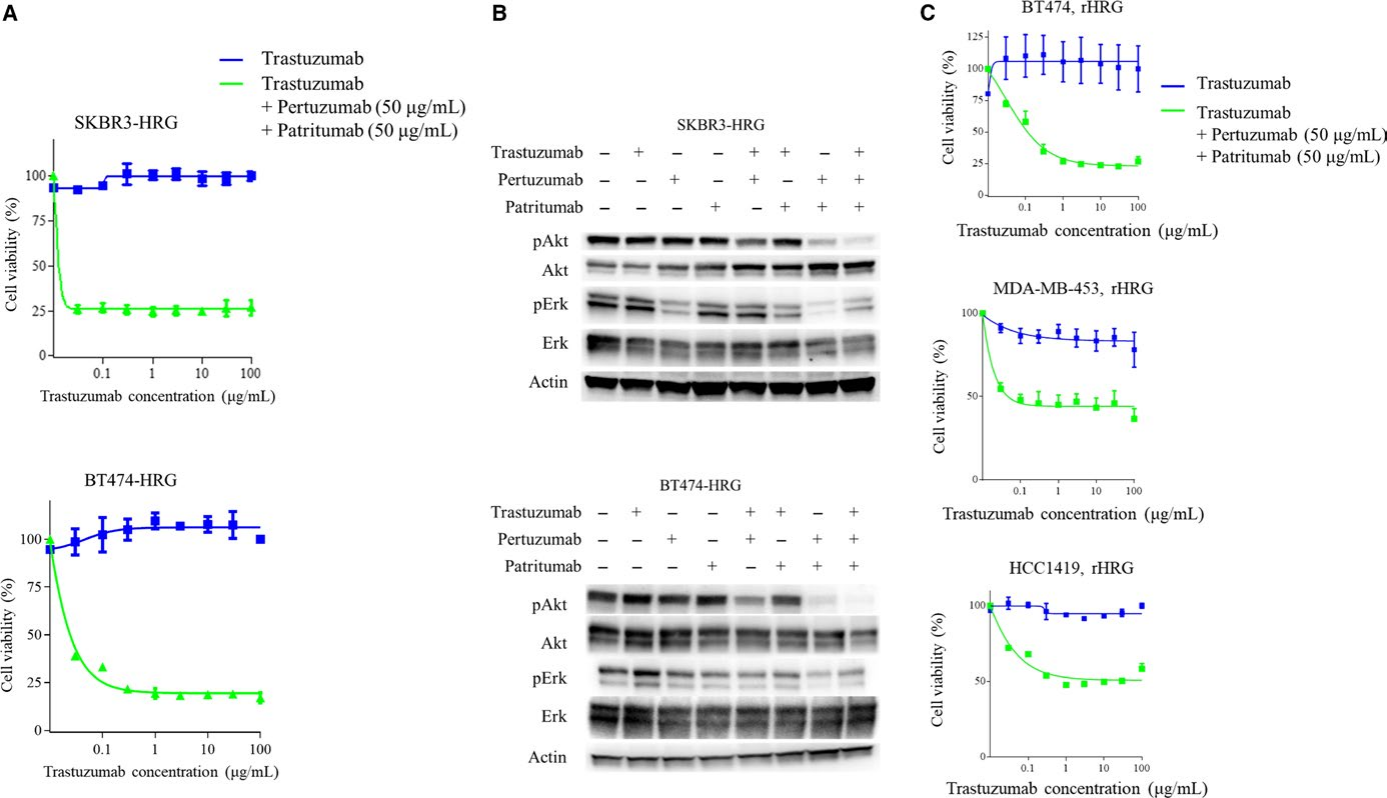
The combination of trastuzumab + pertuzumab + patritumab inhibits cell viability and downstream pAkt in heregulin‐expressing cells. A, SKBR3‐HRG and BT474‐HRG cells were incubated for 120 h with increasing concentrations of trastuzumab and a fixed dose of pertuzumab (50 μg/mL) and patritumab (20 μg/mL); cell viability was then assessed with Cell Counting Kit‐8. Each point represents the mean ± standard error of three independent experiments. B, SKBR3‐HRG and BT474‐HRG cells were incubated for 24 h in RPMI containing 2% FBS, after which cells were treated with or without trastuzumab (20 μg/mL), pertuzumab (20 μg/mL), and patritumab (20 μg/mL) for 1 h. Then, cells were lysed and subjected to immunoblot analysis for pAkt, Akt, pErk, and Erk, with β‐actin serving as a loading control. C, BT474, MDA‐MB‐453, or HCC1419 cells were incubated for 120 h with increasing concentrations of trastuzumab alone, or trastuzumab, pertuzumab (fixed dose; 50 μg/mL), and patritumab (fixed dose; 50 μg/mL) in the presence of recombinant heregulin (20 ng/mL) in the medium. Cell viability was then assessed with Cell Counting Kit‐8. Each point represents the mean ± standard error of three independent experiments

Given that trastuzumab‐based double combination therapy did not show sufficient efficacy toward heregulin‐expressing cancer cells, we examined the triple combination of trastuzumab, pertuzumab, and patritumab using cell viability assays. Specifically, SKBR3‐HRG and BT474‐HRG cells were treated with fixed doses of pertuzumab (50 μg/mL) and patritumab (50 μg/mL), with escalating doses of trastuzumab (0‐100 μg/mL). Notably, triple therapy drastically decreased viable cell counts even at the lowest concentration of trastuzumab for both SKBR3‐HRG and BT474‐HRG cells (Figure 3A). Consistent with these observations, immunoblotting showed that triple combination therapy more potently inhibited Akt activation, compared to that with pertuzumab/trastuzumab or patritumab/trastuzumab combination treatment in both SKBR3‐HRG and BT474‐HRG cells (Figure 3B). Furthermore, combinations of pertuzumab and patritumab, without trastuzumab, potently decreased Akt phosphorylation in these heregulin‐overexpressing cells (Figure 3B). pErk also tended to decrease upon treatment with combinations of pertuzumab and patritumab or triple therapy in SKBR3‐HRG cells; however, this effect was not as obvious in BT474‐HRG cells (Figure 3B).

According to reports, heregulin is excreted from intestinal cells and activates cancer cells in a paracrine manner.^7^, ^22^ Therefore, we evaluated the effect of triple combination therapy using a heregulin paracrine model with HER2‐positive breast cancer cell lines including BT‐474, MDA‐MB‐453, and HCC1419. In a medium containing recombinant heregulin, these cells were treated with escalating doses of trastuzumab alone or that combined with fixed doses of pertuzumab (50 μg/mL) and patritumab (50 μg/mL). BT‐474, MDA‐MB‐453, and HCC1419 cells were sensitive to trastuzumab (data not shown); however, these cells developed resistance in the presence of heregulin (Figure 3C). Moreover, drug resistance was reversed by adding pertuzumab and patritumab to trastuzumab therapy in these paracrine models (Figure 3C). These results suggested that trastuzumab addition to both pertuzumab and patritumab, but not either alone, can potently inhibit Akt activation in heregulin‐expressing, HER2‐positive breast cancer. Therefore, triple therapy demonstrated potent anticancer efficacy in models of autocrine or paracrine heregulin signaling.

### 3.4 Combined pertuzumab and patritumab inhibits SKBR3‐HRG cell viability

**Figure 4:**
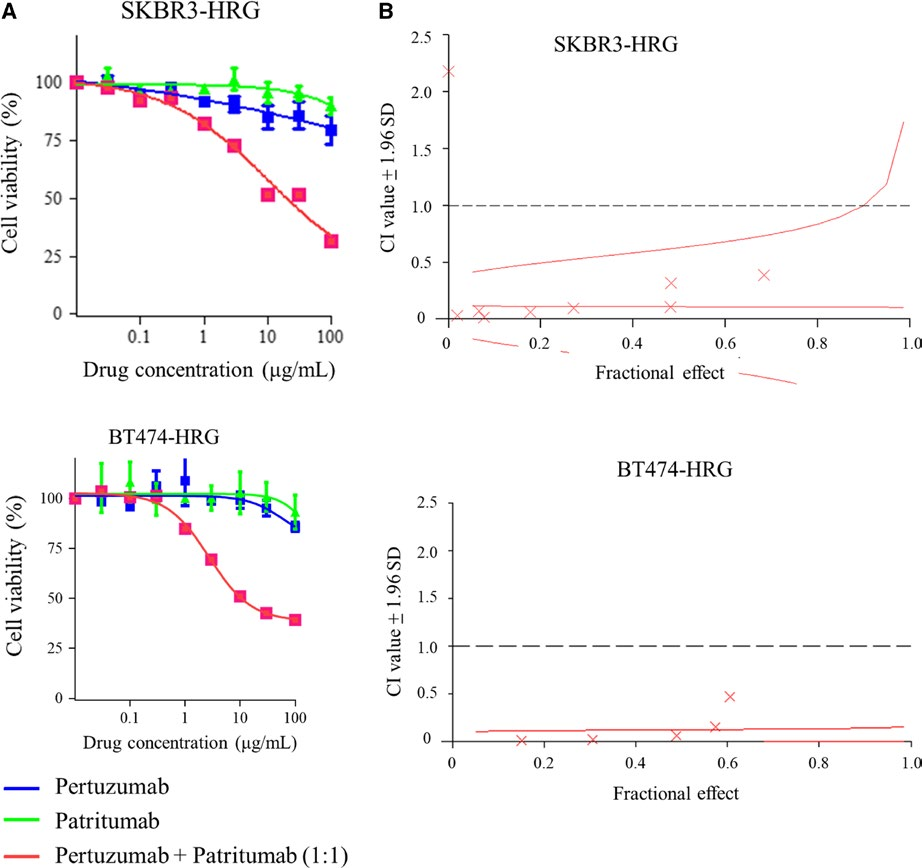
Synergistic effect of pertuzumab and patritumab on heregulin‐expressing cells based on cell viability assays. A, SKBR3‐HRG and BT474‐HRG cells were incubated for 120 h with increasing concentrations of pertuzumab, patritumab, or pertuzumab with patritumab at a 1:1 ratio; cell viability was then assessed with Cell Counting Kit‐8. Each point represents the mean ± standard error of three independent experiments. B, The combination index value was calculated from the results of cell viability assays, as reported in (A), using CalcuSyn. A fractional effect‐combination index plot was then generated. The data shown are the mean of three independent experiments, with the curve showing the range of SDs

Given the results indicating that combined pertuzumab and patritumab could inhibit downstream signaling in heregulin‐expressing cells (Figure 3B), we hypothesized that this combination alone, without trastuzumab, could be effective against these cells. As expected, combination therapy decreased the viability of heregulin‐expressing cells, but pertuzumab or patritumab alone did not (Figure 4A). Furthermore, CI values were calculated based on the results of cell viability assays for combined pertuzumab and patritumab treatments as described in the Materials and Methods (Figure 4B). All CI values were below 1. The CI value of the median effective dose was 0.11 for SKBR3‐HRG and 0.13 for BT474‐HRG cells, indicating the strong synergistic effects of these drugs. The combination of pertuzumab and patritumab was also evaluated in paracrine models including BT‐474, MDA‐MB‐453, and HCC1419 cells supplemented with recombinant heregulin (20 ng/mL). Administration of pertuzumab or patritumab alone did not affect or minimally inhibited cell viability in either cell line supplemented with recombinant heregulin, whereas the combination of these agents suppressed cell viability compared to that with each single agent (Figure S1). This suggested that the combination of pertuzumab and patritumab could be effective for some cancer cells that respond to autocrine or paracrine heregulin. However, it should be noted that based on viability, the efficacy of the triple combination therapy was more apparent in those cell lines.

### 3.5 Combined trastuzumab, pertuzumab, and patritumab inhibits cell proliferation based on clonogenic assays

**Figure 5:**
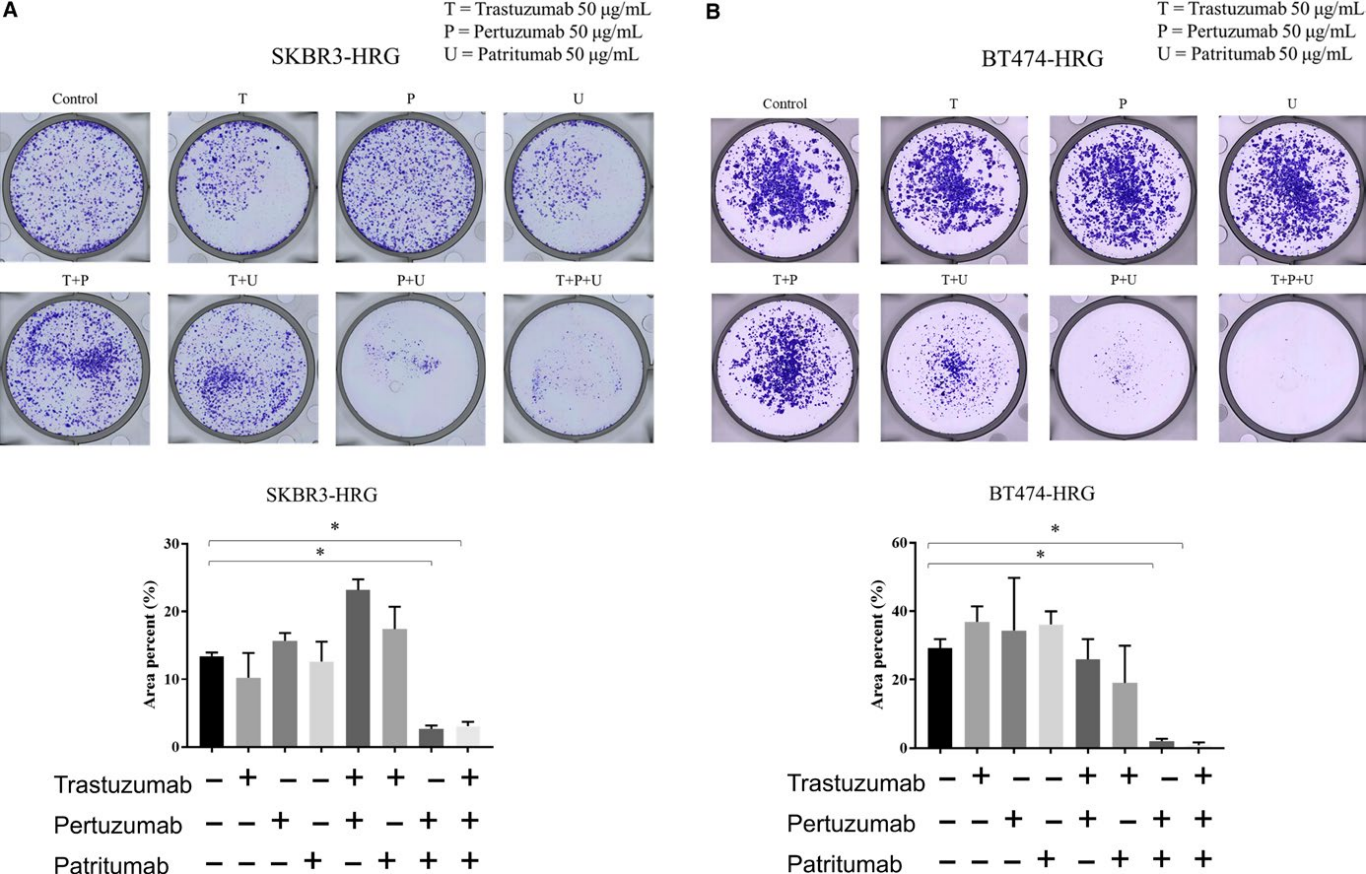
Inhibition of cell proliferation by pertuzumab and patritumab with or without trastuzumab in heregulin‐expressing cells based on clonogenic assays. A and B, SKBR3‐HRG (A) and BT474‐HRG (B) cells were seeded in 6‐well plates and then treated with or without trastuzumab (50 μg/mL), pertuzumab (50 μg/mL), or patritumab (50 μg/mL) for 14 or 17 days. Cells were then fixed and stained with crystal violet. Representative images of clonogenic assays are shown. Total areas of colonies were quantified by ImageJ. Data represent the mean ±standard error of three independent experiments. *P < 0.05 (unpaired *t* test).

Long‐term treatment occasionally causes feedback‐mediated activation of the targeted pathway, which ultimately decreases the anticancer efficacy of such therapuetics.^23^ Considering this possibility, anticancer efficacy was additionally assessed by performing clonogenic assays, in which cells were continually treated for 2 weeks or longer. Consistent with the short‐term effects on cell viability, neither trastuzumab, pertuzumab, nor patritumab inhibited SKBR3‐HRG and BT474‐HRG cell proliferation (Figure 5A,B). Furthermore, pertuzumab or patritumab combined with trastuzumab did not inhibit cell proliferation compared to that in controls (Figure 5A,B). Notably, either the double combination of pertuzumab and patritumab or the triple combination of trastuzumab, pertuzumab, and patritumab significantly inhibited cell proliferation, with the latter showing maximum inhibition of cell proliferation (*P* < 0.05; Figure 5A,B). No statistically significant difference was observed between the specific double and triple combination therapies (*P* = 0.45; Figure 5A,B). In contrast to heregulin‐expressing cells, SKBR3‐Mock and BT474‐Mock cells were sensitive to trastuzumab, but resistant to pertuzumab, patritumab, and those combinations (Figure S2). These results suggested that the antitumor efficacy, based on pertuzumab and patritumab combination, depends on heregulin expression.

### 3.6 In vivo antitumor activity of combined trastuzumab, pertuzumab, and patritumab therapy

**Figure 6:**
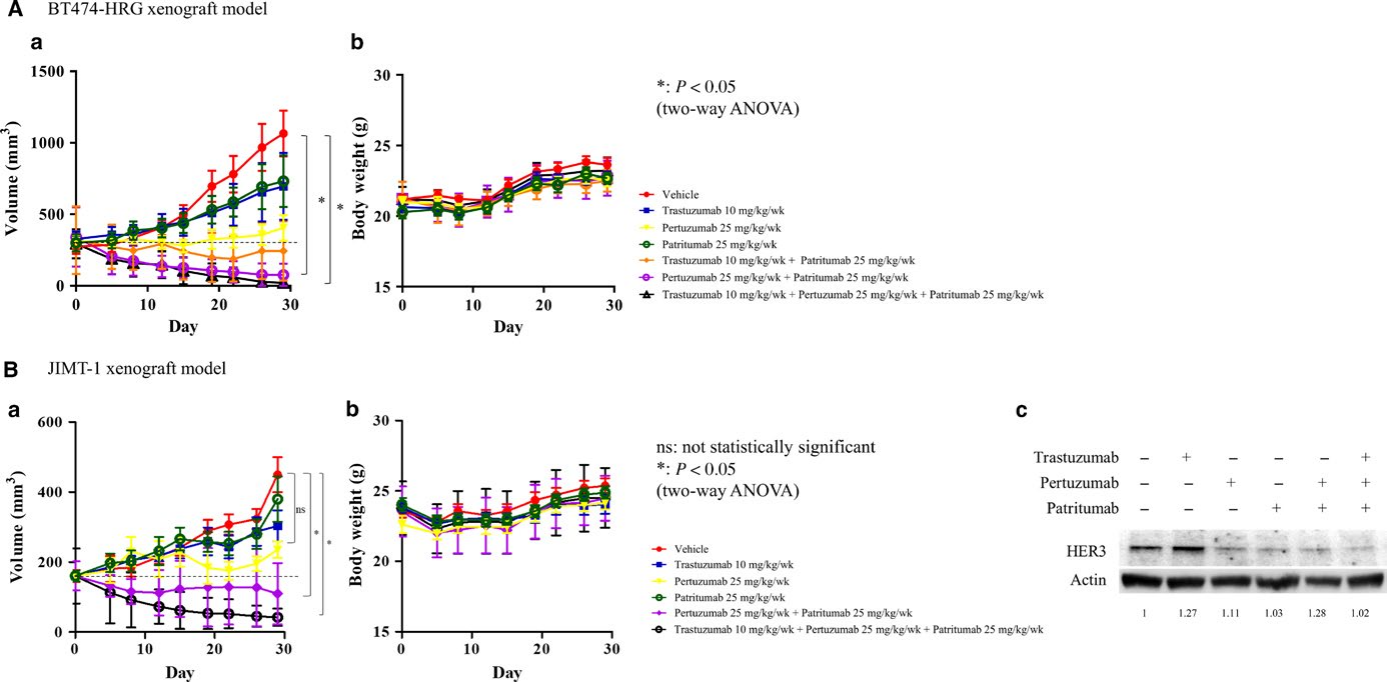
Efficacy of pertuzumab and patritumab with or without trastuzumab on BT474‐HRG and JIMT‐1 tumor xenograft models. A, Vehicle (PBS: 100 μL), trastuzumab (10 mg/kg), pertuzumab (25 mg/kg), patritumab (25 mg/kg), trastuzumab (10 mg/kg) + patritumab (25 mg/kg), pertuzumab (25 mg/kg) + patritumab (25 mg/kg), and trastuzumab (10 mg/kg) + pertuzumab (25 mg/kg) + patritumab (25 mg/kg) were administered via intraperitoneal injection once per week to mice bearing BT474‐HRG cell xenografts. B, Vehicle (PBS: 100 μL), trastuzumab (10 mg/kg), pertuzumab (25 mg/kg), patritumab (25 mg/kg), pertuzumab (25 mg/kg) + patritumab (25 mg/kg), and trastuzumab (10 mg/kg) + pertuzumab (25 mg/kg) + patritumab (25 mg/kg) were administered via intraperitoneal injection once per week to mice bearing JIMT‐1 cell xenografts. Tumor volume (a) and body weights (b) were measured twice per week. Data represent the mean ± standard error. *P < 0.05 (two‐way ANOVA). C, Posttreatment expression of HER2 and HER3 in tumors from xenograft‐bearing mice, as determined by immunoblotting. Cell lysates were analyzed for HER2 and HER3 expression, with β‐actin serving as a loading control.

The combined antitumor effect of trastuzumab, pertuzumab, and patritumab was evaluated in vitro using HRG‐overexpressing cell lines including SKBR3‐HRG and BT474‐HRG cells. However, considering the tumor microenvironment and antibody‐dependent cellular cytotoxicity, we examined the reproducibility of this combined effect in vivo using a tumor xenograft mouse model. Heregulin‐expressing BT474‐HRG xenograft tumors did not shrink in response to trastuzumab alone (Figure 6A). Consistent with our in vitro results, pertuzumab or patritumab alone also did not significantly inhibit tumor growth (Figure 6A). Trastuzumab and patritumab insufficiently prevented cell proliferation in BT474‐HRG cells in vitro, whereas this combination partially prevented tumor growth in vivo. Finally, pertuzumab +patritumab and especially trastuzumab + pertuzumab + patritumab significantly prevented tumor growth and mediated tumor regression (vs vehicle; *P* < 0.05; Figure 6A). Furthermore, trastuzumab + pertuzumab + patritumab completely eradicated three of seven tumors (Figure 6A). These results were consistent with in vitro observations and suggested that a combination of three antibodies might be beneficial.

BT474‐HRG cells are virus‐transduced heregulin‐expressing cells, which might be associated with detrimental effects of these cells. Considering this, we also investigated endogenous heregulin‐expressing cancer cells using a xenograft mouse model. JIMT‐1, a HER2‐positive breast cancer cell line, was previously reported to overexpress heregulin.^24^ Consistent with the BT474‐HRG xenograft model, treatment with pertuzumab and patritumab and especially trastuzumab, pertuzumab, and patritumab induced significant tumor regression compared to that in controls, with the maximum effect induced by the triple combination therapy (*P* < 0.05; Figure 6B). These treatments were also well‐tolerated, as evidenced by the negligible reduction in body weight (<5% of initial weight; Figure 6A,B). In addition, immunogenic assays showed that trastuzumab increased HER3 expression, whereas pertuzumab, patritumab, pertuzumab/patritumab, and trastuzumab/pertuzumab/patritumab suppressed HER3 expression (Figure 6C).

In summary, the HER3 ligand heregulin possibly mediates resistance to trastuzumab in HER2‐positive breast cancer. However, triple trastuzumab/pertuzumab/patritumab therapy might overcome this resistance in these tumors.

## 4 DISCUSSION

The current study identified the synergistic anticancer effect of combined anti‐HER3 patritumab and anti‐HER2 pertuzumab for heregulin (HER3 ligand)‐expressing HER2‐positive breast cancer. Furthermore, heregulin mediated resistance to trastuzumab in these breast cancer cell lines, whereas triple therapy including trastuzumab, pertuzumab, and patritumab demonstrated potent antitumor efficacy. Considering those observations, we suggest that triple blockade of HER2/HER3 signaling could overcome heregulin‐mediated resistance to trastuzumab in HER2‐positive breast cancer. Heregulin expression and its significance with respect to agents that target the HER2‐HER3 axis should be examined with clinical samples.

Previously, we reported that heregulin contributes to trastuzumab resistance in HER2‐positive gastric cancers in a preclinical model.^14^ Those observations are most likely due to the inability of trastuzumab to inhibit ligand‐dependent HER2‐HER3 interactions.^14^ Intriguingly, among patients with gastric cancer, a subpopulation with high plasma heregulin expression was associated with shorter survival after paclitaxel plus trastuzumab treatment, compared to that in a subpopulation with low heregulin.^25^ In contrast, there was no difference in survival between those subpopulations when patients were treated with paclitaxel alone. Although it could not be confirmed that circulating heregulin was derived from the tumor, this ligand potentially mediates resistance to trastuzumab in various types of cancer. Thus, triple therapy with trastuzumab/pertuzumab/patritumab might provide optimal efficacy for multiple types of ligand‐dependent and HER2‐positive tumors including breast and gastric cancer.

Consistent with the antitumor synergistic effect, anti‐HER2 pertuzumab and anti‐HER3 patritumab combination therapy inhibited AKT signaling more potently than each antibody alone. However, we could not fully elucidate the underlying mechanism for this effect. Garrett et al reported that dual HER2 inhibition with lapatinib and trastuzumab increases HER3 cell surface expression in BT474 and SKBR3 cells.^26^ Additionally, inhibition of HER2/PI3K/AKT has been shown to induce the upregulation of HER3 mediated by FOXO.^27^, ^28^ Consistently, our current study demonstrated HER3 induction upon HER2 inhibition by trastuzumab or pertuzumab in BT474‐HRG cells (Figure S3). Therefore, HER3 upregulation via HER2 inhibition might enhance the efficacy of patritumab.

Alternatively, HER2 or HER3 inhibition alone might not sufficiently prevent the activation of ligand‐dependent HER2‐HER3‐AKT signaling. Garette et al reported that trastuzumab plus the anti‐HER2 pertuzumab or trastuzumab plus the anti‐HER3 seribantumab prevents heregulin‐driven cancer cell proliferation, whereas this efficacy was lost with excessive heregulin concentrations (>1 nmol/L).^26^ Dual inhibition of HER2‐HER3 interactions by different mechanisms might have the advantage of robustly preventing heregulin‐dependent HER2‐HER3‐AKT signaling. Further studies are warranted to elucidate the mechanism associated with this synergistic interaction between pertuzumab and patritumab in heregulin‐expressing HER2‐positive breast cancer.

In the current study, we examined the effect of combination therapy in vivo using the heregulin‐overexpressing BT474‐HRG xenograft model. Trastuzumab alone, pertuzumab alone, or patritumab alone could not significantly prevent tumor growth, whereas triple therapy resulted in tumor reduction. Furthermore, triple therapy had no effect on body weight in BT474‐HRG xenograft mice. However, the fact that we overexpressed heregulin could not exclude possible detrimental effects on drug sensitivity; therefore, we utilized an additional mouse model based on the endogenous heregulin‐expressing HER2‐positive breast cancer cell line JIMT‐1.^24^ Consistent with that observed with the BT474‐HRG xenograft model, triple combination therapy induced tumor regression and had no effect on body weight in this model. Similarly, a previous report demonstrated that trastuzumab/lapatinib/patritumab could improve survival compared to that with trastuzumab/lapatinib in a BT474 xenograft model.^26^

Many therapeutic agents targeting HER3 have demonstrated promising results in preclinical studies, whereas the clinical use of those agents has not resulted in meaningful benefits.^12^, ^15^, ^18^, ^19^ In contrast, the U3‐1402 anti‐HER3 antibody (patritumab) conjugated with topoisomerase demonstrated impressive efficacy with a 48% response rate in patients with HER3‐positive pretreated breast cancer.^29^ However, some patients were still refractory to U3‐1402 in this early‐phase clinical trial.^29^ Hereafter, combination strategies might improve the efficacy of U3‐1402 against these HER3‐positive tumors. The current study demonstrated that HER2 and HER3 dual blockade with trastuzumab, pertuzumab, and patritumab potently inhibits Akt activation, upon which cancer cells are dependent for the evasion of apoptosis. This dual inhibition also resulted in potent anticancer effects in conditions of enhanced autocrine or paracrine heregulin signaling. Considering these results, U3‐1402 plus pertuzumab should be examined to determine whether this treatment can potently prevent AKT activation, and to assess its synergistic anticancer efficacy compared to that with U3‐1402 alone.

## CONFLICT OF INTERESTS

The authors declare that they have no conflict of interest.
