# Supplementary figures and images for: Targeting of the HER2/HER3 signaling axis overcomes ligand‐mediated resistance to trastuzumab in HER2‐positive breast cancer
Source: Cancer Med. 2019 Jan 31;8(3):1258–68. doi: 10.1002/cam4.1995 (PMC6434202; doi:10.1002/cam4.1995)

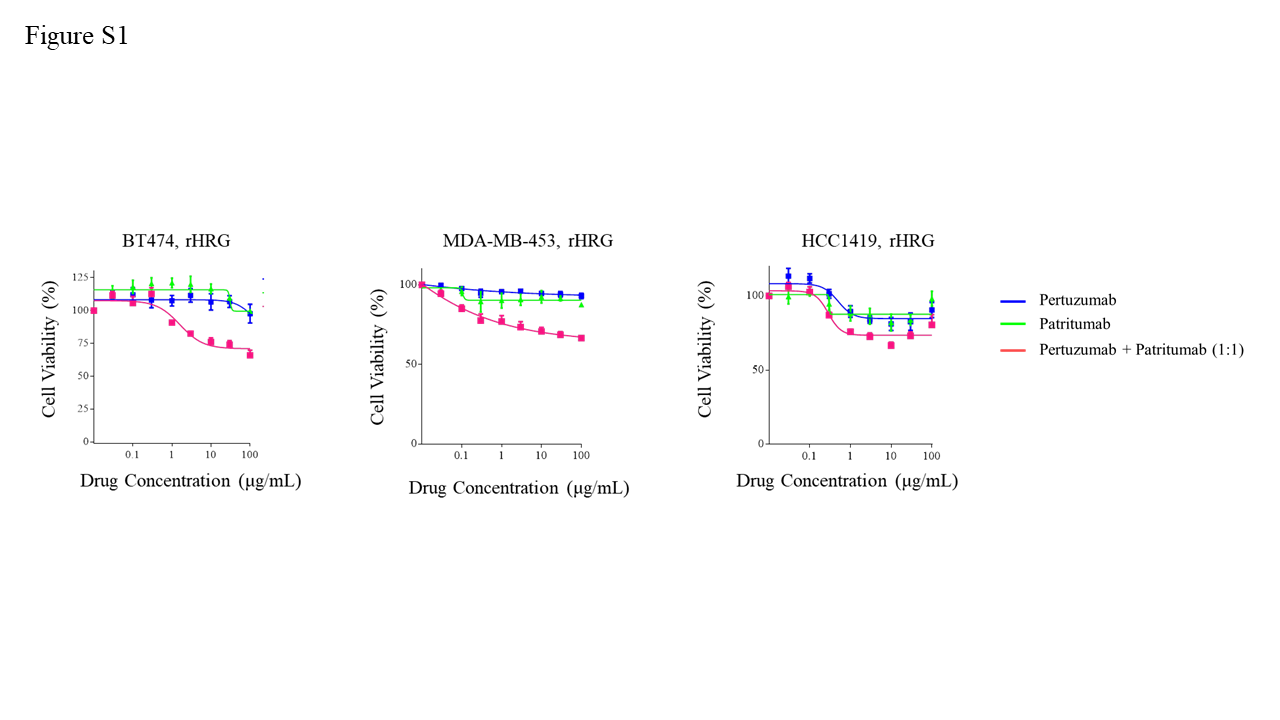

Supplement: Supplementary file 1 [file CAM4-8-1258-s001.tif]

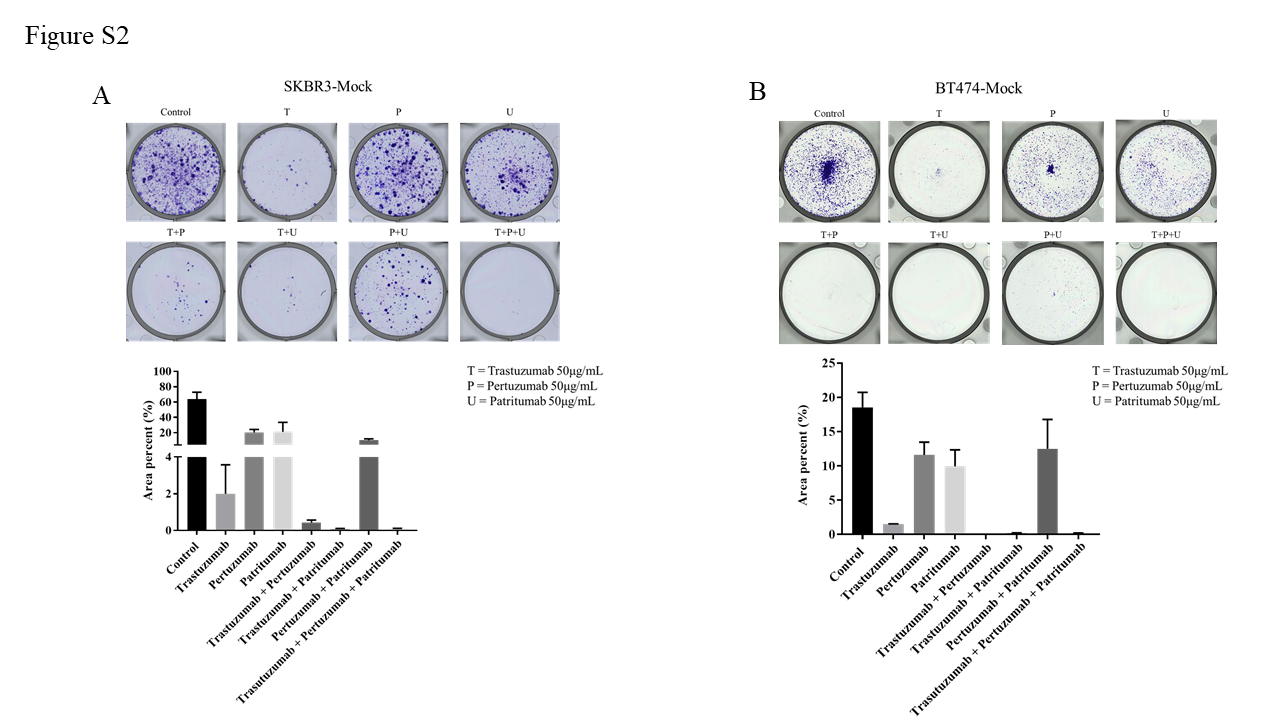

Supplement: Supplementary file 2 [file CAM4-8-1258-s002.tif]

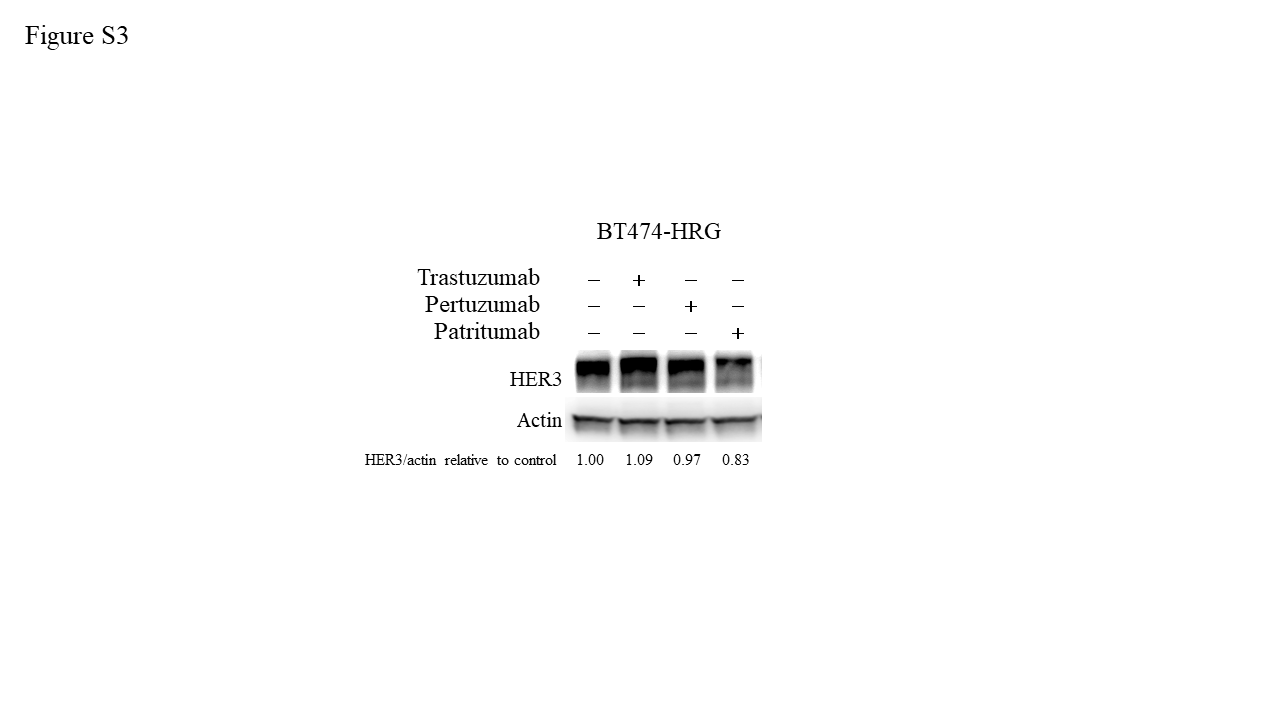

Supplement: Supplementary file 3 [file CAM4-8-1258-s003.tif]
